# Supplementary material for: Force-Matching-Based Approach for the Generation of Polarizable and Nonpolarizable Force Fields Applied to CsPbI3
Source: J Phys Chem C Nanomater Interfaces. 2025 Jan 30;129(6):3040–53. doi: 10.1021/acs.jpcc.4c04979 (PMC11831672; doi:10.1021/acs.jpcc.4c04979)
Supplement: Supplementary file 1 — jp4c04979_si_001.pdf [file jp4c04979_si_001.pdf]

Force-Matching-Based Approach for the Generation of Polarizable and Nonpolarizable Force Fields Applied to $CsPbI_3$

Cecilia Vona, Mathias Dankl, Ariadni Boziki, Martin P. Bircher, and Ursula Rothlisberger*

Laboratory of Computational Chemistry and Biochemistry, Ecole Polytechnique Fédérale de Lausanne (EPFL), CH-1015 Lausanne, Switzerland

E-mail: ursula.roethlisberger@epfl.ch

Phone: +41 (0)21 6930321. Fax: +41 (0)21 930320

AMOEBA force field

For completeness, we explicitly give here the matrix elements of the multipole interaction matrix of the AMOEBA FF, needed to describe the electrostatic interactions between multipoles on sites i and j, respectively:[1]

$$\mathrm{T}_{ij} = \frac{1}{r_{ij}}, \tag{1}$$

$$\mathrm{T}_{ij,\beta} = \nabla_\beta T_{ij}^{\mathrm{D}} = -\lambda_3 \frac{r_{ij,\beta}}{r_{ij}^3}, \tag{2}$$

$$\mathrm{T}_{ij,\beta\gamma} = \nabla_\beta T_{ij,\gamma} = \lambda_5 \frac{3 r_{ij,\beta} r_{ij,\gamma}}{r_{ij}^5} - \lambda_3 \frac{\delta_{\beta\gamma}}{r_{ij}^3}, \tag{3}$$

$$\mathrm{T}_{ij,\beta\gamma\eta} = \nabla_\beta T_{ij,\gamma\eta} = \lambda_7 \frac{15 r_{ij,\beta} r_{ij,\gamma} r_{ij,\eta}}{r_{ij}^7} + \lambda_5 \frac{3(r_{ij,\beta}\delta_{\gamma\eta} + r_{ij,\gamma}\delta_{\beta\eta} + r_{ij,\eta}\delta_{\beta\gamma})}{r_{ij}^5}. \tag{4}$$

$$
\mathrm{T}_{ij,\beta\gamma\eta\nu} = \nabla_\beta T_{ij,\gamma\eta\nu} = \lambda_9 \frac{105 r_{ij,\beta} r_{ij,\gamma} r_{ij,\eta} r_{ij,\nu}}{r_{ij}^9} -
$$

$$
\lambda_7 \frac{15(r_{ij,\beta} r_{ij,\gamma}\delta_{\eta\nu} + r_{ij,\beta} r_{ij,\eta}\delta_{\gamma\nu} + r_{ij,\beta} r_{ij,\nu}\delta_{\gamma\eta} + r_{ij,\gamma} r_{ij,\eta}\delta_{\beta\nu} + r_{ij,\gamma} r_{ij,\nu}\delta_{\beta\eta} + r_{ij,\eta} r_{ij,\nu}\delta_{\beta\gamma}}{r_{ij}^7} + \quad (5)
$$

$$
\lambda_5 \frac{3(\delta_{\beta\gamma}\delta_{\eta\nu} + \delta_{\beta\eta}\delta_{\gamma\nu} + \delta_{\gamma\nu}\delta_{\beta\eta})}{r_{ij}^5}.
$$

$\beta,\ \gamma,\ \eta$ and ν are Cartesian coordinates and the constant $1/4\pi\varepsilon_0$ is omitted for simplicity. The coefficients λ_t for $t \in \{3,5,7,9\}$ have been introduced by Thole.[2] They are needed to damp the small-range interactions. In the case of permanent multipoles the coefficients λ_t are simply 1, while for the induced multipoles, λ_t are defined as follows:

$$
\lambda_3 = 1 - \exp(-au^3), \tag{6}
$$

$$
\lambda_5 = 1 - (1 + au^3)\exp(-au^3), \tag{7}
$$

$$
\lambda_7 = 1 - \left(1 + au^3 + \frac{3}{5}a^2u^6\right)\exp(-au^3), \tag{8}
$$

$$
\lambda_9 = 1 - [1 + au^3 + (18a^2u^6 + 9a^3u^9)/35]\exp(-au^3). \tag{9}
$$

in which, as mentioned in the main text, $u = r_{ij}/(\alpha_i\alpha_j)^{1/6}$ is the effective distance as a function of atomic polarizabilities α_i and α_j and a is the damping factor.

Bader charges

The Bader charges for the atoms Cs, Pb and I, computed for each DFT trajectory are reported in Table S1. Despite the different phases, cell sizes and temperature conditions, the values of the charges for each atomic species only vary in the order of 10^{-2} e, more specifically the range of variation is 0.039 e for Cs, 0.037 for Pb and 0.0183 for I. Additionally, within the

Table S1: Bader charges per atomic species for all the α and δ phase DFT trajectories generated at different temperatures and unit cell sizes (for V_0, V_1 look Table 1). The last row shows the average.

Phase	Size	T [K]	q_{Cs} [e]	q_{Pb} [e]	q_I [e]
α	V_0	650	0.840 ± 0.002	0.921 ± 0.002	-0.587 ± 0.001
α	V_1	650	0.853 ± 0.006	0.920 ± 0.003	-0.591 ± 0.003
δ	V_0	100	0.8208 ± 0.0006	0.947 ± 0.001	-0.5892 ± 0.0003
δ	V_0	300	0.8174 ± 0.0007	0.943 ± 0.001	-0.5869 ± 0.0002
δ	V_0	500	0.814 ± 0.001	0.937 ± 0.003	-0.5837 ± 0.0008
δ	V_1	100	0.851 ± 0.002	0.967 ± 0.002	-0.606 ± 0.001
δ	V_1	300	0.846 ± 0.002	0.961 ± 0.003	-0.602 ± 0.001
δ	V_1	500	0.840 ± 0.001	0.952 ± 0.005	-0.597 ± 0.001
Average			0.839 ± 0.015	0.936 ± 0.018	-0.592 ± 0.007

same trajectories, the atomic charges undergo only small oscillations, leading to standard deviations of at most of 0.006 e. From these data, we computed the averaged atomic Bader charges discussed in the text. The average has been weighted according to the inverse number of trajectories for each phase (1/2 for α and 1/6 for δ).

Radial pair distribution at 0K

Comparing the radial pair distribution functions (RPDFs) of the structures optimized with the *npol* and *pol* model with the experimental structure and the DFT optimized geometry, we found a good agreement for the α phase of $CsPbI_3$ (Figure S1). The same is not observed for the δ phase, for which the *npol* model shows discrepancies for the pairs Pb-I, Pb-Pb and Cs-Cs (Figure 1 in the manuscript). For the pairs I-I, Cs-I and Pb-Cs, the deviations, even when present, are hardly visible (Figure S2).

Cell size at finite temperatures

In Table S2, we report the average volume per s.u. of the NPT trajectories for the non-melting systems. In parenthesis, we show the percentage deviation from the experimental

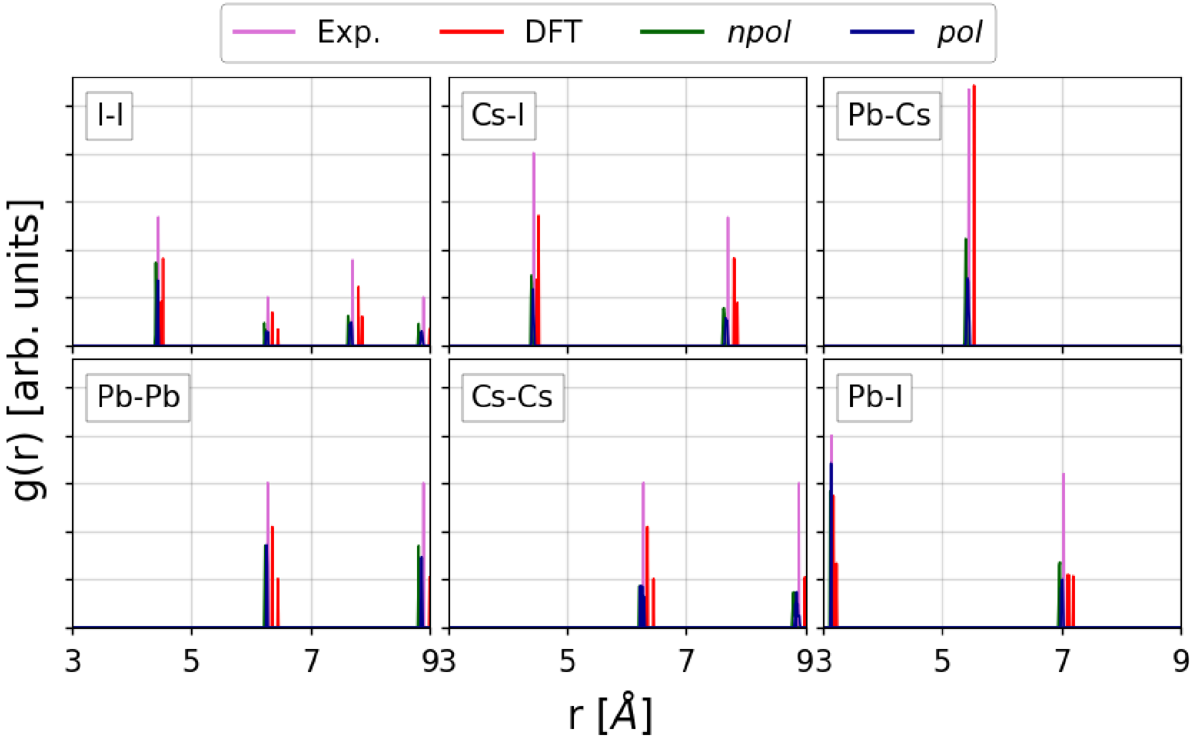

Figure S1: RPDFs of the α phase computed for the experimental structure[3] and the structures optimized with DFT and the force fields (FFs) *npol* and *pol*.

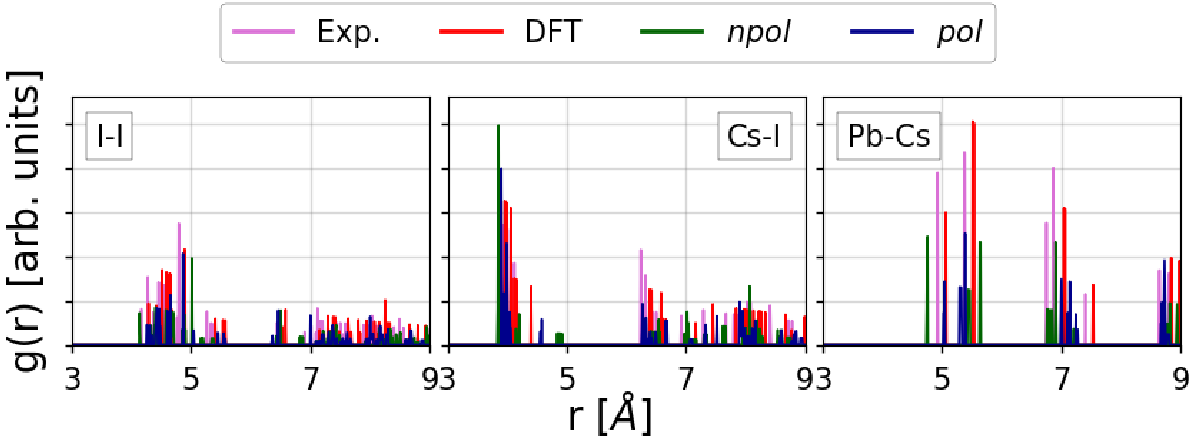

Figure S2: RPDFs of the δ phase for the experimental structure[4] and for structures optimized with different methods. In the figure, the RPDFs are shown only for the pairs I-I, Cs-I and Pb-Cs. Those of the other pairs are in the main text.

structure (see Table 1). The axes from which the volumes are computed oscillate anisotropically. We observe that the average volumes of trajectories generated with the *npol* model expand slightly more rapidly with increasing temperature than that of the trajectories gen-

erated with *pol*. Moreover, by comparing the deviation of the DFT structure from the experimental structure, also in Table 1, we can observe that the volumes of the trajectories at high temperature (600 K - 650 K) are comparable to those of the V_1 structure (21% for the α phase and 26% for the δ phase). In this respect, it is convenient to compare the properties of the high-temperature trajectories to those of the V_1 DFT reference.

Table S2: Average cell volumes (in Å^3 per s.u.) at different temperatures of the NPT classical trajectories. In parenthesis, we include the relative deviation with respect to the experimental structure volume (see Table 1).

	α-phase		δ phase	
	npol	*pol*	*npol*	*pol*
100 K	244.0 ± 0.4 (-2%)	243.8 ± 0.4 (-2%)	236.8 ± 0.5 (7%)	233.8 ± 0.4 (5%)
300 K	257.2 ± 0.8 (3%)	255.4 ± 0.7 (3%)	250.9 ± 0.9 (13%)	245.6 ± 0.8 (11%)
500 K	275 ± 1 (11%)	270 ± 1 (8%)	274 ± 2 (24%)	262 ± 2 (18%)
600 K	286 ± 2 (15%)	-	-	-
650 K	297 ± 2 (19%)	-	-	-

Optimized α phase cell from the 100K trajectory

In Table S3, we show the axes length and the volume of the α phase cells optimized using as a starting point a frame from the NPT 100K trajectories. In both cases, the axes show that the cells are no longer perfectly cubic. Also, the octahedra exhibits some tilting. In comparison to the cells optimized using the experimental structure as the starting point, the volume is smaller. The larger deviation is for the *pol* model, for which the relative deviation with respect to the experimental structure becomes -4%, while for the cell optimized from the experimental structure it is -1% (Table 1).

Radial pair distribution functions at finite temperatures

In this section, we discuss the RPDFs at finite temperatures of α phase NVT trajectories and δ phase NVT and NPT trajectories. The RPDFs for the α phase NPT trajectories and

Table S3: α phase axes lengths, average cell volumes and relative deviation with respect to the experimental structure of the supercell optimized with the *npol* and *pol* model starting from a frame of the respective NPT 100K trajectory.

	npol	*pol*
a [Å]	43.48	43.57
b [Å]	43.33	43.27
c [Å]	43.42	43.42
V per s.u.[$Å^3$]	238.5	238.7
ΔV %	-4%	-4%

for the δ phase NPT trajectories for T = 100 K, are shown and discussed in the main text. In Figure S3, we show the RPDFs of the α phase NVT trajectories using the *npol* and *pol* FFs. The DFT references (V_0 and V_1) are from the trajectories at 650K. The peaks of the RPDFs of the classical trajectories are slightly shifted to the left with respect to the DFT reference V_0, which is as well shifted to the left with respect to those of the DFT V_1 trajectory. The reason is that when optimizing the experimental α phase[3] with the *npol* and *pol* models, the volume of the cell contracts while the DFT relaxation leads to a structure with expanded volume, which is additionally expanded in the V_1 system. By comparing the RPDFs of the classical trajectories for constant volume, we observed that with increasing temperature, the position of the peak is kept fixed but the broadening of the peaks increases. Finally, the differences between the RPDFs of the *npol* and *pol* models are small and hardly visible by comparing the plots.

Contrarily, for the δ phase (Figures S4-S10), the poorer agreement of the *npol* model with the DFT reference is evident. The differences are a consequence of the discrepancies observed in δ phase structure optimized with the *npol* model, which are visible in the RPDFs at 0K (Figures 1-S2). Due to the increase of the broadening at higher temperatures, the disagreement reduces with increasing temperature but remains evident for the Cs-Cs pairs.

The *pol* model shows a very good agreement with the DFT references. In particular, the RPDFs of the NPT trajectories up to 300 K and all the NVT trajectories agree well with the DFT V_0 reference, while the RPDFs of the NPT trajectories at higher temperature agree

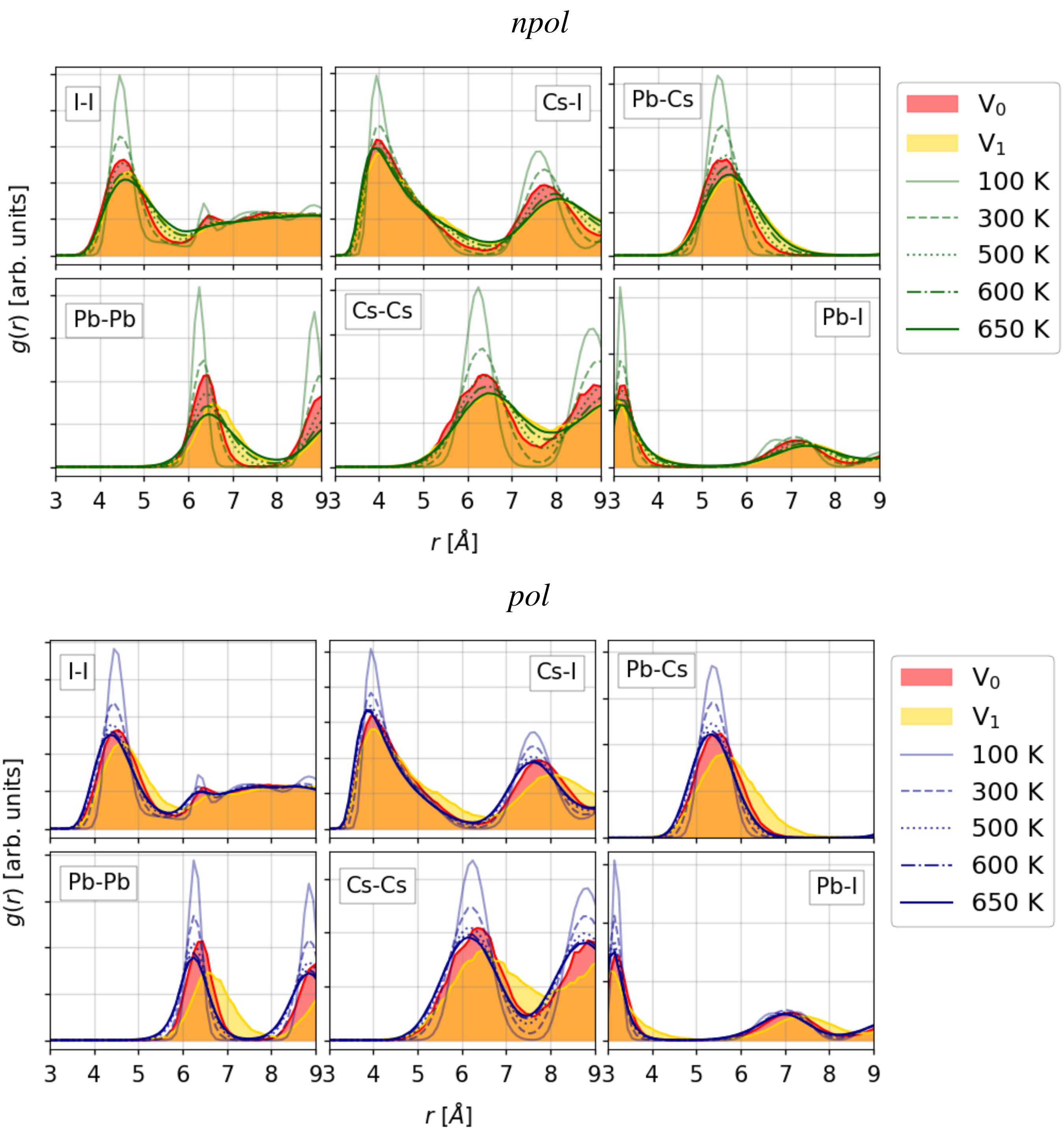

Figure S3: RPDFs of the α phase trajectories generated at finite temperature in an NVT ensemble. In the top panel, the RPDFs for the *pol* trajectories are shown while in the bottom panel, those for the *npol* trajectories are given. The temperature range is 100-650 K. The curves are compared with the DFT references at 650K.

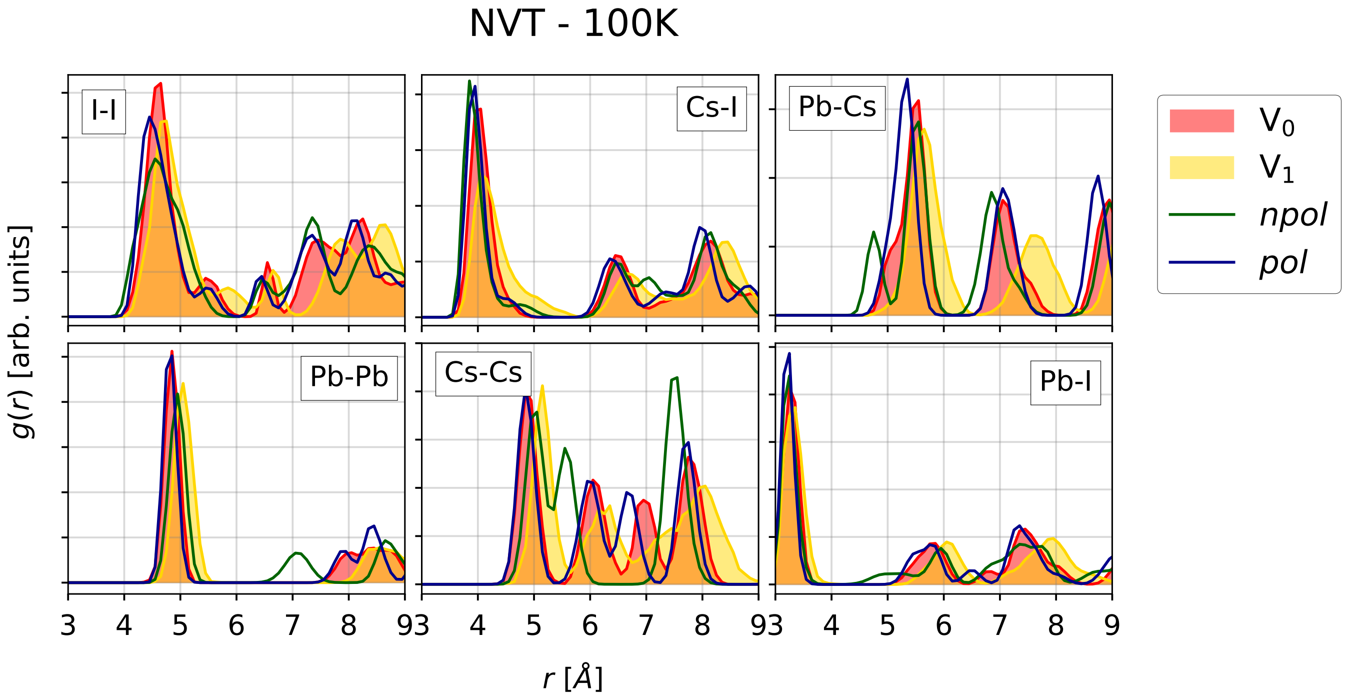

Figure S4: RPDFs of δ phase trajectories generated in an NVT ensemble at 100 K with the *npol* and *pol* FFs. The DFT trajectories, V_0 and V_1, are also generated at 100 K.

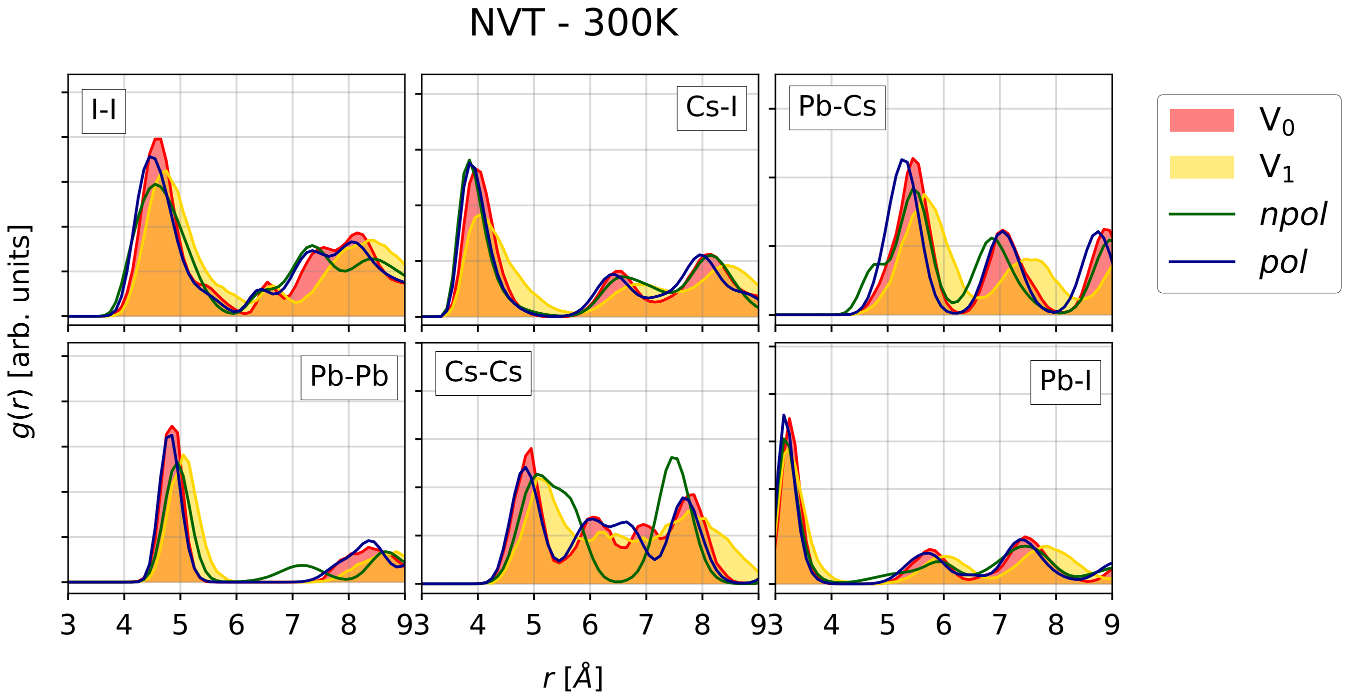

Figure S5: RPDFs of δ phase DFT and classical trajectories at 300 K. The classical trajectories are generated in an NVT ensemble.

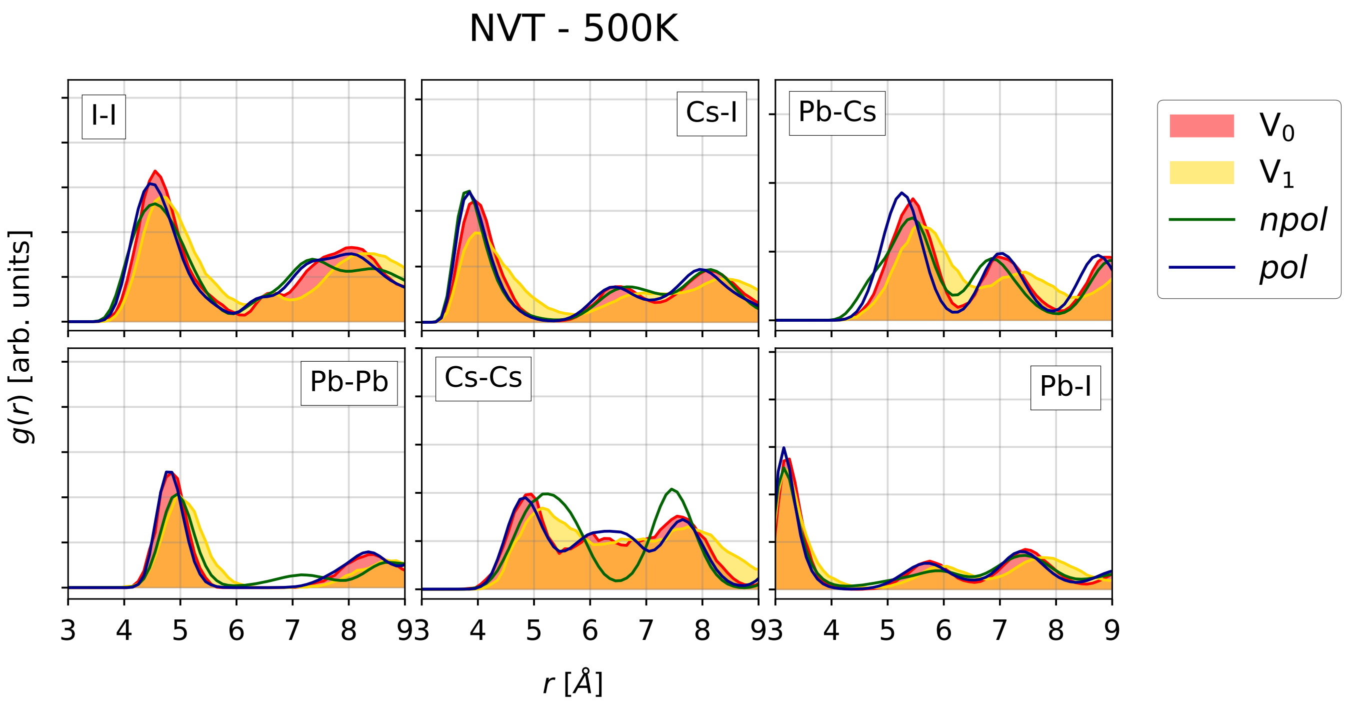

Figure S6: RPDFs of δ phase DFT and classical trajectories at 500 K. The classical trajectories are generated in an NVT ensemble.

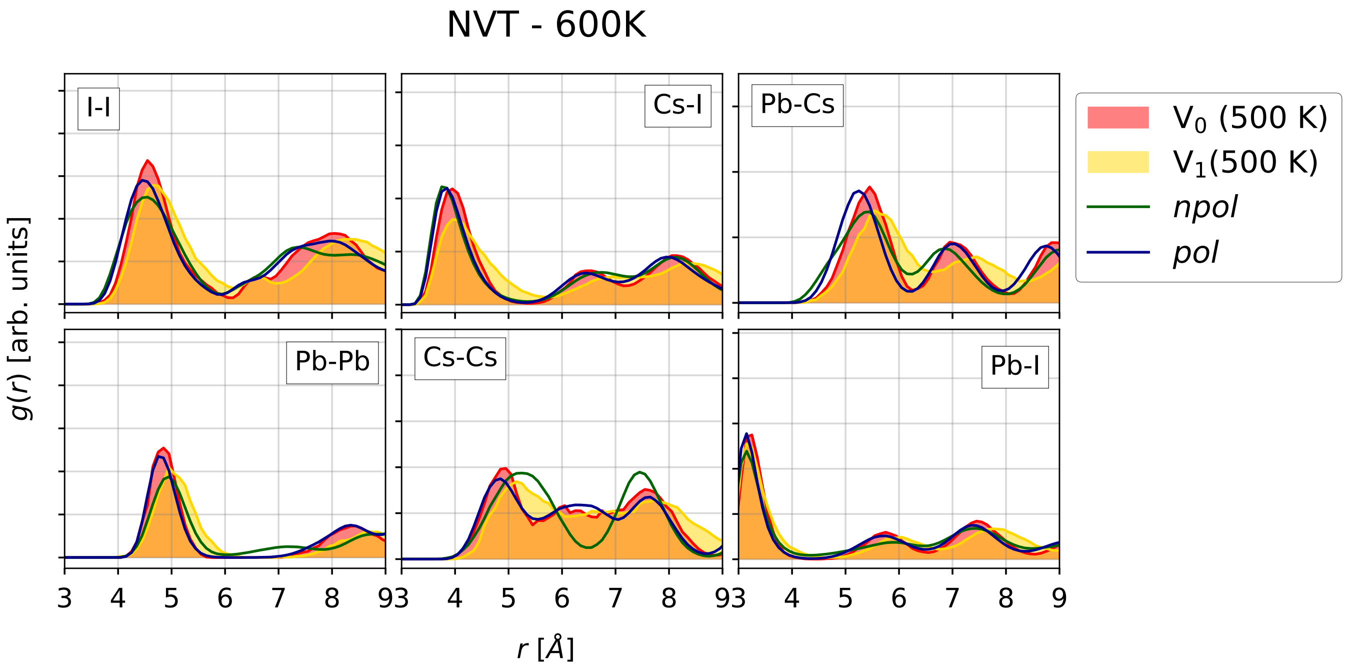

Figure S7: RPDFs of δ phase trajectories. The classical trajectories are generated in an NVT ensemble at 600K and DFT trajectories at 500K.

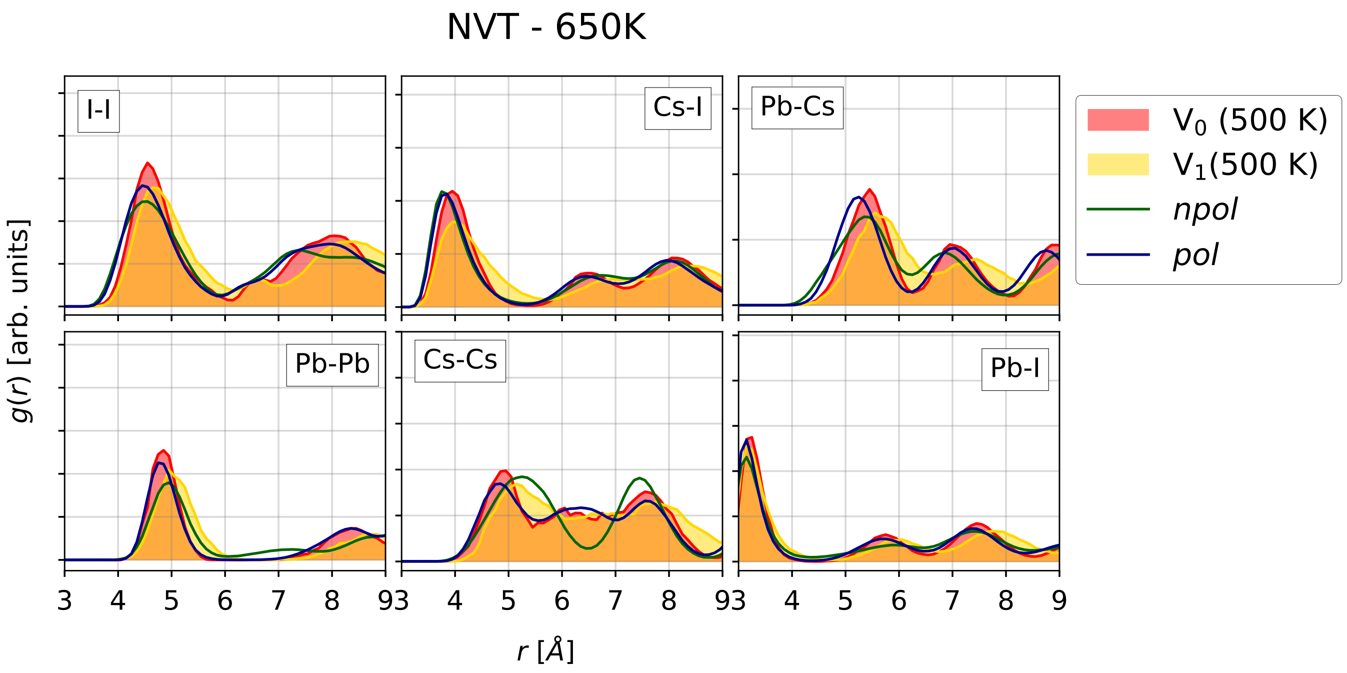

Figure S8: RPDFs of δ phase trajectories. The classical trajectories are generated in an NVT ensemble at 650K and DFT trajectories at 500K.

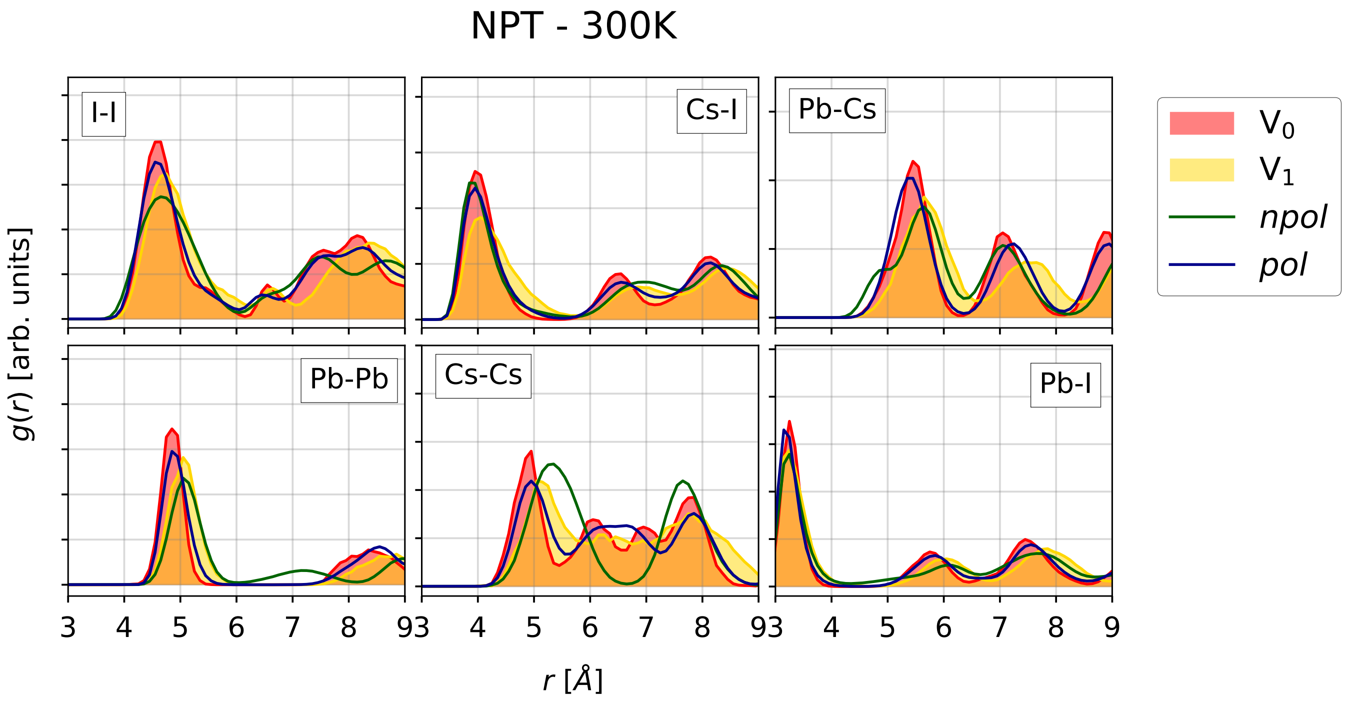

Figure S9: RPDFs of δ phase classical and DFT trajectories at 300 K. The classical trajectories are generated in an NPT ensemble.

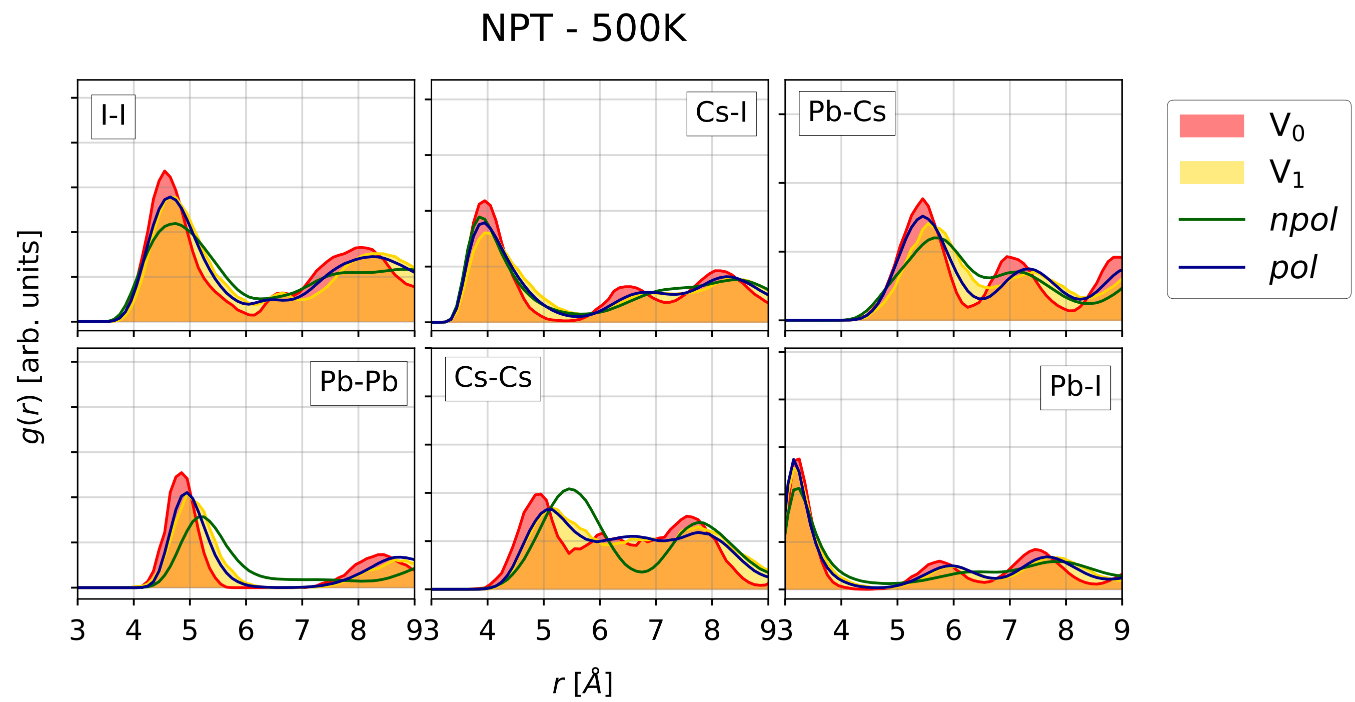

Figure S10: RPDFs of δ phase classical and DFT trajectories at 500 K. The classical trajectories are generated in an NPT ensemble.

better with DFT V_1. The reason can be found in the volume expansion discussed in the previous section.

Power spectra

In Figures S11 and S12, the atom resolved power spectra of the δ and α phase of classical trajectories generated in NVT ensemble at different temperatures are plotted. For all spectra, by increasing the temperature there are no distinct features and the spectra are relatively continuous. The peak positions do not vary when increasing the temperature. Moreover, at all the temperatures, for all the atoms there are major differences between the two phases. In Figures S13-S14, we show the total power spectra.

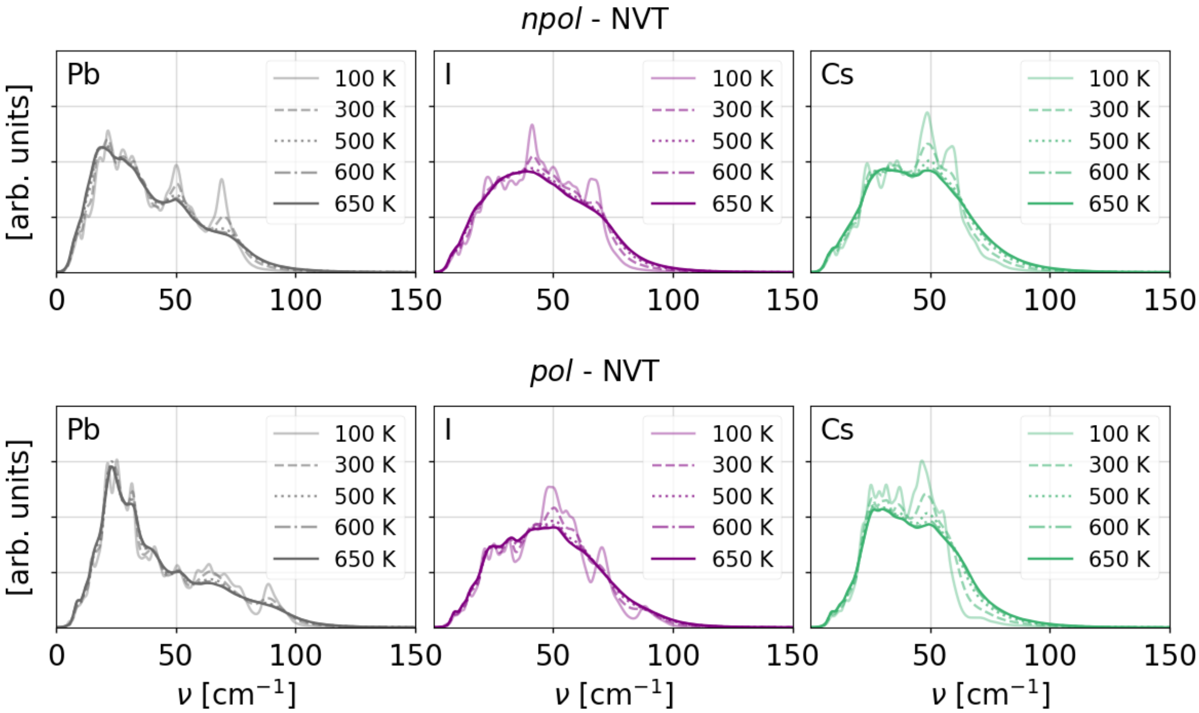

Figure S11: Species projected power spectra of δ phase NVT trajectories generated with the *npol* and *pol* model at different temperatures.

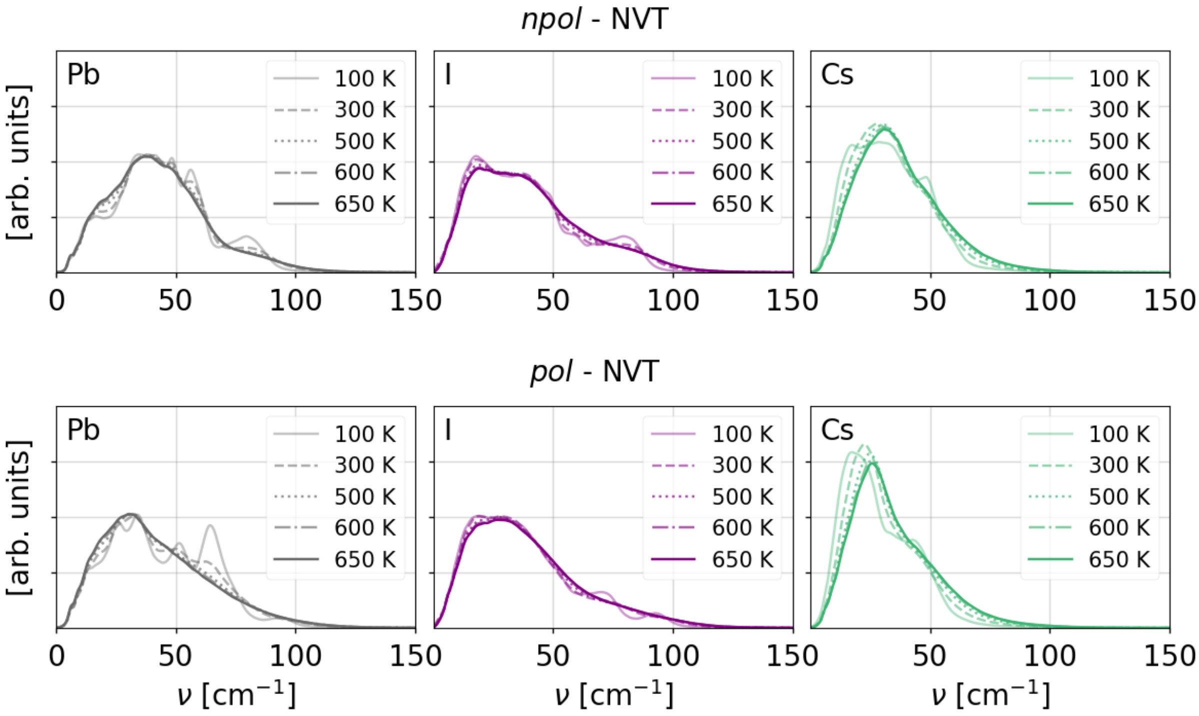

Figure S12: Species projected power spectra of α phase NVT trajectories generated with the *npol* and *pol* model at different temperatures.

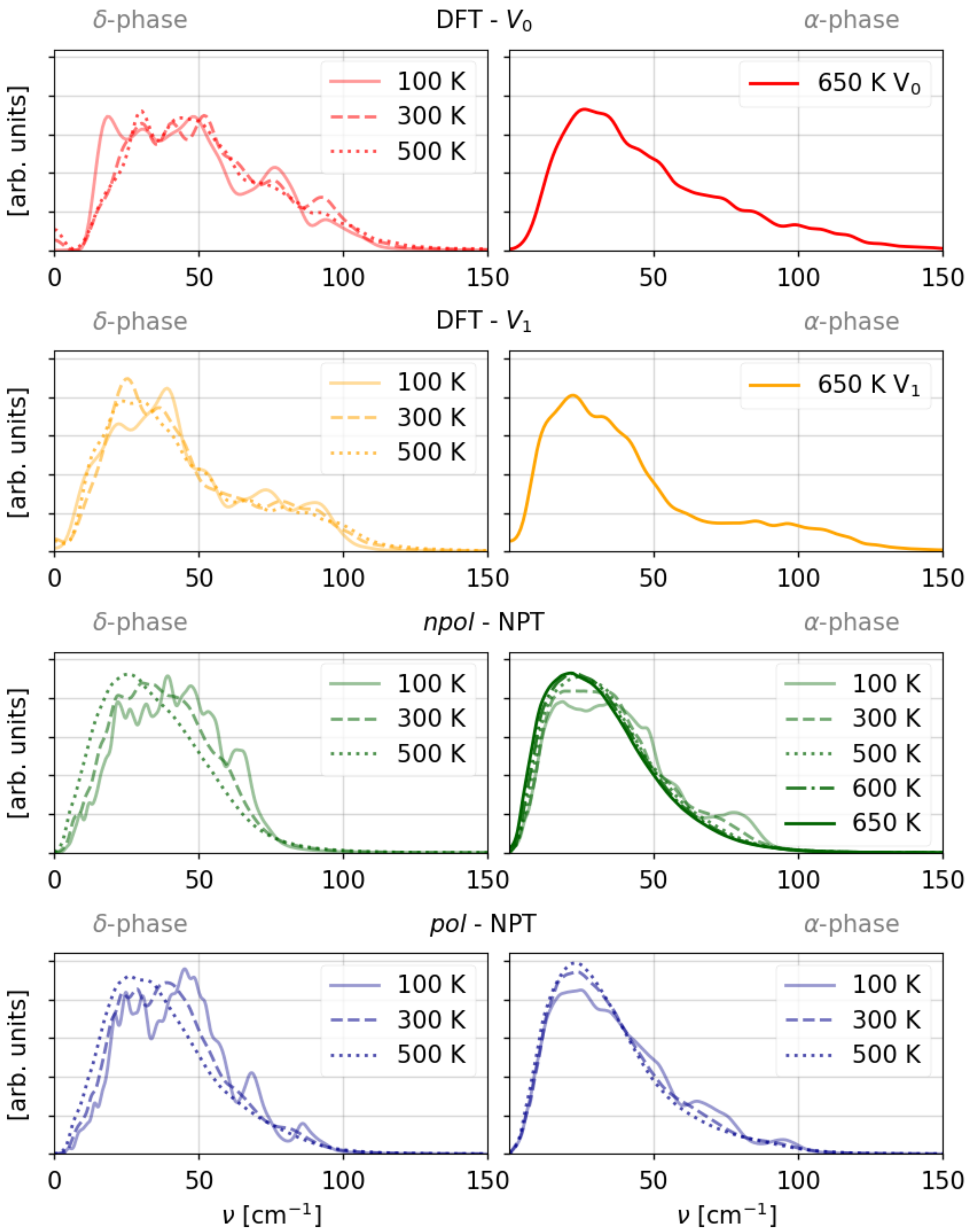

Figure S13: Total power spectra of the δ phase (left panels) and α phase (right panel) $CsPbI_3$ trajectories. The first two rows show the DFT power spectra for the unit cell size V_0 and V_1 (see Table 1), and the last two show the power spectra from NPT trajectories generated with the *npol* and *pol* model at different temperatures.

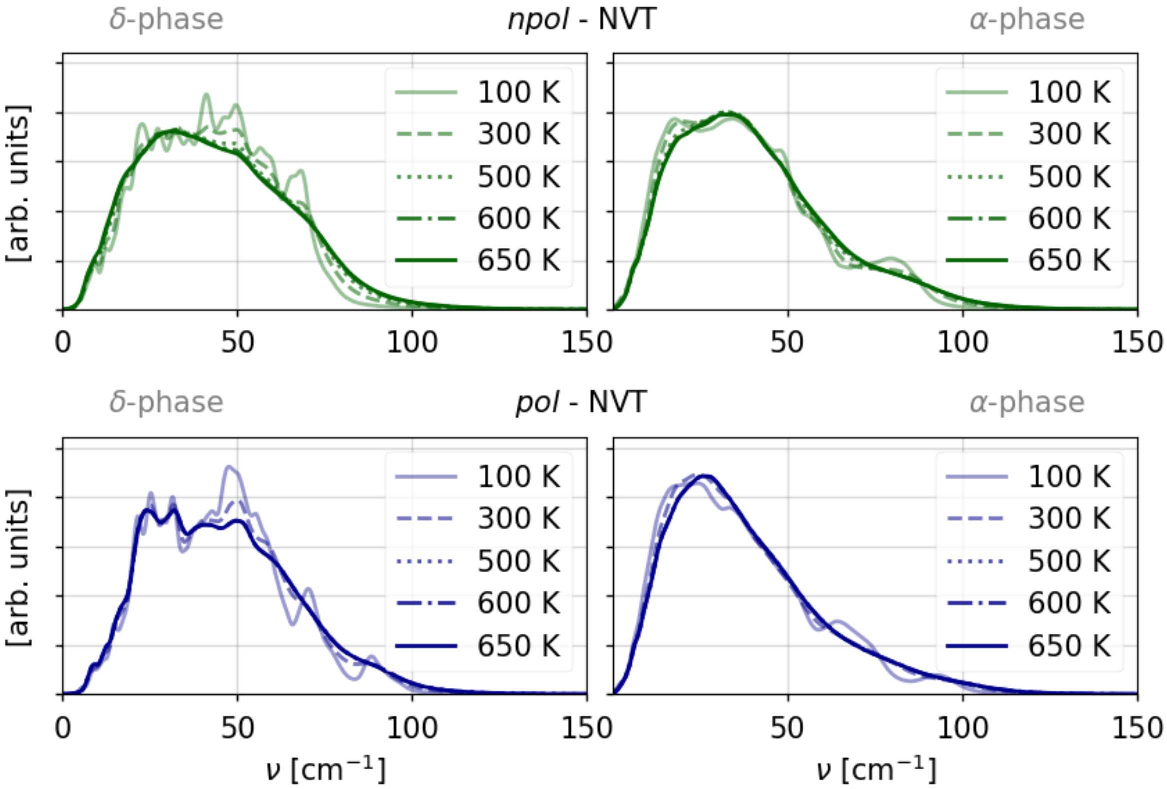

Figure S14: Total power spectra of NVT classical trajectories of the δ phase (left panels) and α phase (right panel) generated with the *npol* and *pol* model at different temperatures.

Energies at finite temperatures

In Figure S15, there are the plots of the total energy and individual energy contributions of the NVT classical trajectories generated with the *npol* and *pol* FFs. As in the case of NPT trajectories, analyzed in the main text, the energetic order in the *npol* simulations invert already at 100 K, while in the *pol* trajectories, no inversion occurs but the relative energy difference reduces with increasing temperature. In the NVT simulations, the vdW contribution plays an important role for the energetic ordering and looks quite different with respect to the NPT case.

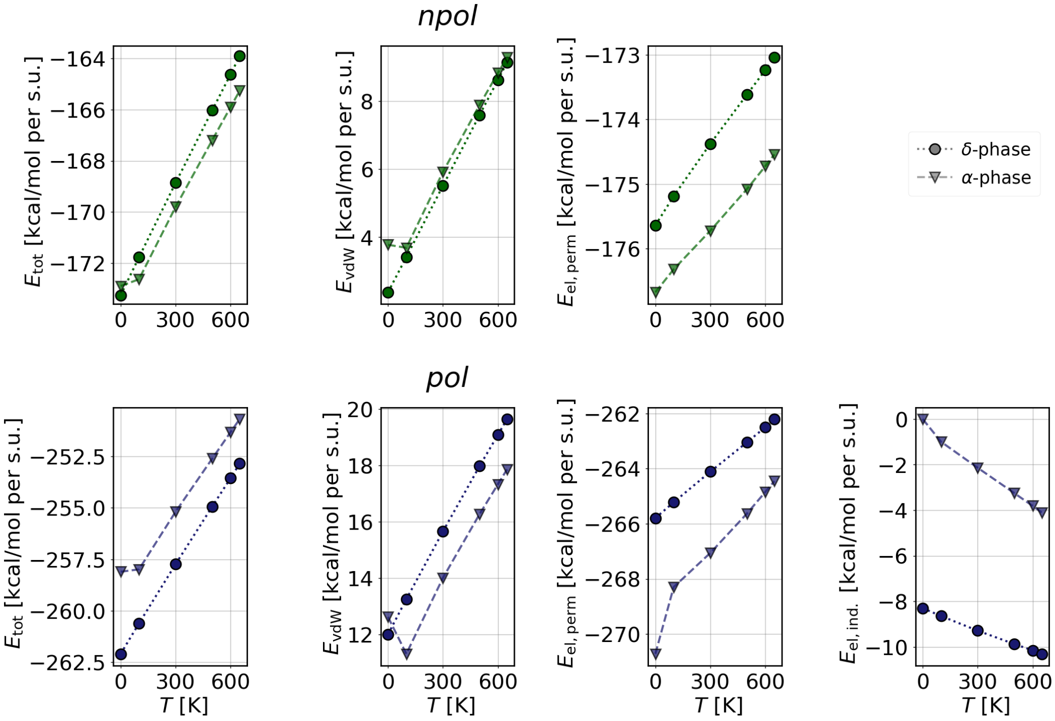

Figure S15: Energies of the classical NVT trajectories at finite temperatures of the δ and α phase of $CsPbI_3$. For the *npol* FFs in addition to the total energies, there are only the vdW and permanent electrostatic contribution, while for the *pol* FF also the induced electrostatic contribution is plotted.

Convergence tests

In Tables S4-S6 and Fig. S16 we report some of the most relevant convergence tests.

Table S4: Convergence of the total energy difference for the wave function cutoff (`ecutwfc`) in QE using a cutoff of 70 Ry as reference.

`ecutwfc` (Ry)	ΔE_{tot} (Ry per s.u.)
30	9.4×10^{-3}
40	1.8×10^{-3}
50	9.8×10^{-4}
60	1.6×10^{-4}

Table S5: Convergence behavior of the total energy difference and the maximum difference in force component for the k-points grid for δ phase performed with QE. The values are obtained using a $6 \times 6 \times 6$ k-grid as reference.

k-points	ΔE_{tot} (Ry per s.u.)	$\max\{\Delta F_{i.\alpha}\}$ (Ry/a.u.)
$1 \times 1 \times 1$	1.7×10^{-1}	1.3×10^{-2}
$1 \times 3 \times 1$	7.4×10^{-4}	1.3×10^{-3}
$2 \times 4 \times 1$	2.1×10^{-4}	1.9×10^{-4}
$2 \times 4 \times 2$	1.4×10^{-4}	1.6×10^{-4}

Table S6: Total energy cutoff benchmark in CPMD. ΔE_{tot} is evaluated using the values for $E_{\mathrm{cut}} = 120$ Ry as reference.

E_{cut} (Ry)	ΔE_{tot} (Ry per s.u.)
50	6.2×10^{-3}
70	4.4×10^{-4}
90	1.8×10^{-4}
110	3.9×10^{-5}

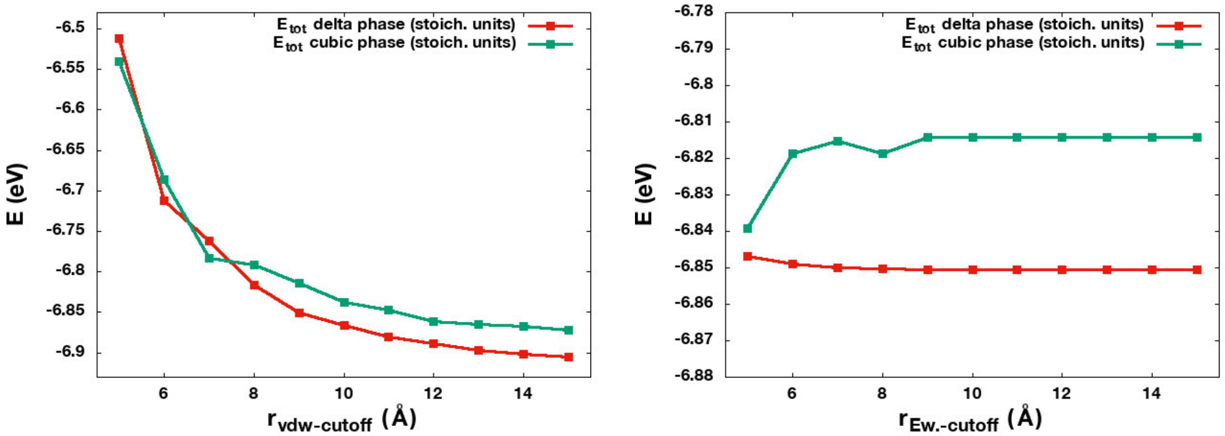

Figure S16: Example of vdW and Ewald cutoff convergence behavior. The final parameters have been chosen using a convergence criteria of $\sim 10^{-3}$ Ry per s.u. $= 1.4 \times 10^{-2}$ eV per s.u..

References

(1) Ren, P.; Ponder, J. W. *The Journal of Physical Chemistry B* **2003**, *107*, 5933–5947.

(2) Thole, B. *Chemical Physics* **1981**, *59*, 341–350.

(3) Trots, D.; Myagkota, S. *Journal of Physics and Chemistry of Solids* **2008**, *69*, 2520–2526.

(4) Stoumpos, C. C.; Malliakas, C. D.; Peters, J. A.; Liu, Z.; Sebastian, M.; Im, J.; Chasapis, T. C.; Wibowo, A. C.; Chung, D. Y.; Freeman, A. J.; Wessels, B. W.; Kanatzidis, M. G. *Crystal Growth & Design* **2013**, *13*, 2722–2727.
